# Rapid HPLC Quantification Approach for Detection of Active Constituents in Modern Combinatorial Formula, San-Huang-Xie-Xin-Tang (SHXXT)

**DOI:** 10.3389/fphar.2016.00374

**Published:** 2016-10-20

**Authors:** Tung-Ying Wu, Fang-Rong Chang, Jing-Ru Liou, I-Wen Lo, Tang-Chia Chung, Li-Yao Lee, Chun-Chen Chi, Ying-Chi Du, Man-Hon Wong, Suh-Hang Hank Juo, Chun-Chen Lee, Yang-Chang Wu

**Affiliations:** ^1^Chinese Medicine Research and Development Center, China Medical University HospitalTaichung, Taiwan; ^2^Graduate Institute of Natural Products, College of Pharmacy, Kaohsiung Medical UniversityKaohsiung, Taiwan; ^3^Center for Infectious Disease and Cancer Research, Kaohsiung Medical UniversityKaohsiung, Taiwan; ^4^Cancer Center, Kaohsiung Medical University HospitalKaohsiung, Taiwan; ^5^Research Center for Environmental Medicine, Kaohsiung Medical UniversityKaohsiung, Taiwan; ^6^Department of Pharmacy, Kaohsiung Medical University HospitalKaohsiung, Taiwan; ^7^Graduate Institute of Medical Genetics, College of Medicine, Kaohsiung Medical UniversityKaohsiung, Taiwan; ^8^School of Pharmacy, College of Pharmacy, China Medical UniversityTaichung, Taiwan; ^9^Research Center for Chinese Herbal Medicine, China Medicinal UniversityTaichung, Taiwan; ^10^Center for Molecular Medicine, China Medical University HospitalTaichung, Taiwan

**Keywords:** HPLC, San-Huang-Xie-Xin-Tang (SHXXT), *Coptis chinensis*, *Rheum officinale*, *Rheum tanguticum*, *Scutellaria baicalensis*, quantification, quality control

## Abstract

San-Huang-Xie-Xin-Tang (SHXXT), one of the most important traditional Chinese medicinal formulas, is comprised by three herbal medicines, the rhizome of *Rheum officinale* [or *Rheum tanguticum* (Polygonaceae) (Dahuang in Chinese)], the root of *Scutellaria baicalensis* (Labiatae) (Huangqin in Chinese), and the rhizome of *Coptis chinensis* (Ranunculaceae) (Huanglian in Chinese) in the ratios of 2:1:1 or 1:1:1. This study is aimed to quantitate and qualify of SHXXT, by a rapid, convenient, and effective HPLC-PDA approach associated with LC-MS technique. Of which method, nine chosen major bioactive components in SHXXT, including aloe-emodin (**Ale**), baicalin (**Ba**), berberine (**Be**), coptisine (**Co**), palmatine (**Pa**), resveratroloside (**Res**), rhein (**Rh**), sennoside A (**Se-A**), and wogonin (**Wo**), were evaluated within 30 min. The nine chemical markers were monitored in a high sensitivity with a low detection limit of 0.01−0.55 μg/mL and the correlation coefficient of the regression curve revealed a good linearity with *R*^2^ > 0.99. Moreover, the extraction solution system and the HPLC elution conditions were also optimized in the present study. This present developed protocol was then successfully applied to quantify nine chemical markers of 10 SHXXT products from eight Taiwanese TCM pharmaceutical companies. In quantitative results, **Res** was found as the major compound in SHXXT-1~5 and 8 with significantly higher amounts than those in other products, indicating the products SHXXT-1~5 and 8 may use *R. tanguticum* as the raw material, which possessed a higher concentration of the bioactive composition **Res**, instead of *R. officinale*. Simultaneously, **Ale**, **Rh**, and **Wo** were < 2% in these 10 products. Different chemical profiles of commercial products indicated that, probably, each product with the same named formula might be regarded as a sole medicine and need to be investigated individually. Importantly, it is never too much to emphasize the importance of quality control in TCM development.

## Introduction

In Asian countries, traditional Chinese medicines (TCM) are widely used for improving the human health. Traditionally, medicinal herbs are usually prescribed as the form of combinatorial formula based on the traditional medicinal philosophy, and most herbal medicines are administered as decoctions (pronounced “Tang” in Chinese; Li S. L. et al., 2010). However, the complicated processes of extract preparation and problematic manufacture procedures cause difficulties in the production of therapeutically equivalent medicines or other convenient pharmaceutical dosage forms (Sheridan et al., 2012). Although modernized extraction approach may help overcome some problems, current clinical evidences are still insufficient to ensure the therapeutic equivalence of herbal decoctions, indicating that a single marker would not define the quality of TCM (Sheridan et al., 2012). Importantly, the chemical marker ingredients of TCM are commonly used to standardize a herbal extract while the complex TCM formula with various ingredients complicate the quality control. Therefore, to develop an appropriate and efficient method in quality control of the combinatorial formula of TCM is an essential and critical issue, especially for the pharmaceutical manufacturers.

San-Huang-Xie-Xin-Tang (SHXXT), one of the most important traditional Chinese medicinal formulas, is comprised by three herbal medicines, the rhizome of *Rheum officinale* Baill. [or *Rheum tanguticum* Maxim. et Balf (Polygonaceae) (Dahuang in Chinese)], the root of *Scutellaria baicalensis* Georgi. (Labiatae) (Huangqin in Chinese), and the rhizome of *Coptis chinensis* Franch. (Ranunculaceae) (Huanglian in Chinese) in the ratios of 2:1:1 or 1:1:1. According to the well-known and classical Chinese medicine ancient books, jinkui yaolue (the golden chamber), the use of SHXXT is to dry dampness, remove toxicity, and purge heat. The indications are constipation, swelling, and pain of body, especially in the eyes, yellow and greasy tongue coating heat accumulation and choking sensation in chest, forceful and rapid pulse, sores, boils, aphtha, and hypertension. In addition, the traditional administration was to decoct the three materials in water for oral dose to be taken twice. Nowadays, SHXXT was produced and sold as Chinese patent medicine. Due to the inconsistency of manufacturing process in different CGMP-TCM biotechnical companies, the composition, quantity, and even the dosage were all have to be based on the instruction sheet of each product.

**Figure 1:**
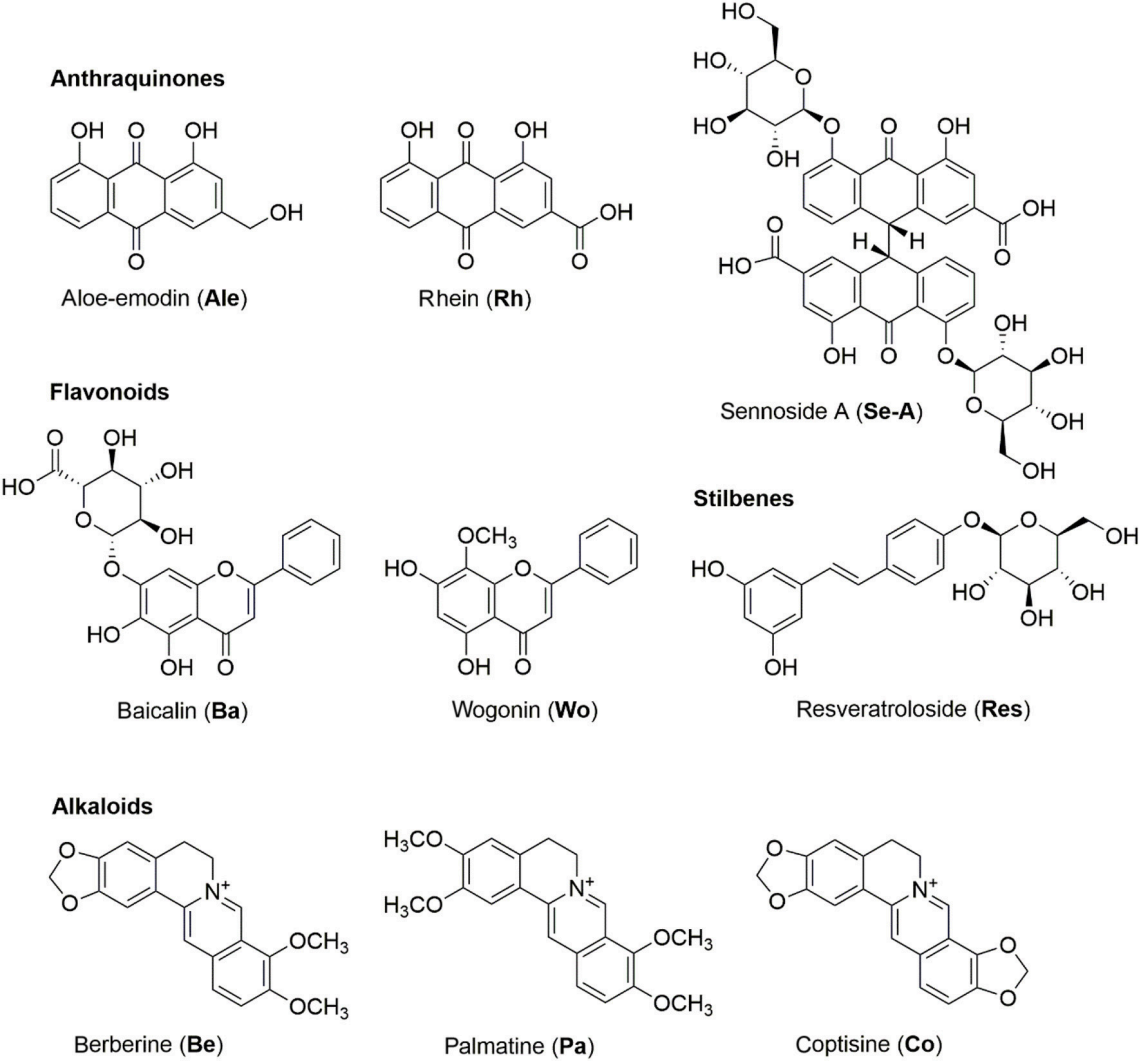
**The structures of target anthraquinones, stilbenes, flavonoids, and alkaloids of SHXXT**.

In the modern scientific literatures, the pharmacological activities of SHXXT revealed its potential for symptoms of gastrointestinal (GI) disorders, such as gastritis, gastric bleeding, peptic ulcers, and abnormal GI motility (Kim et al., 2014; Hwang et al., 2015), anti-hypertension (Tsai et al., 2008), anti-inflammatory (Shih et al., 2007), neuroprotection (Lo et al., 2012), anti-atherogenic (Wang Y. S. et al., 2011), anti-oxidant (Shia et al., 2011), immunomodulatory (Li C. Y. et al., 2010), anti-cancer (Cheng et al., 2008), gastrointestinal tract diseases (Saegusa et al., 2003), and cardiovascular disease (Liou et al., 2012). In the last decades, the phytochemical investigations of herbal medicines found stilbenes and anthraquinones in *R. tanguticum*, flavonoids in *S. baicalensis*, and alkaloids in *C. chinesis* act as the major components and these compounds were therefore chosen to be the biomarkers in quality control. The structures of nine bioactive compounds, aloe-emodin (**Ale**), baicalin (**Ba**), berberine (**Be**), coptisine (**Co**), palmatine (**Pa**), resveratroloside (**Res**), rhein (**Rh**), sennoside A (**Se-A**), wogonin (**Wo**), are shown in Figure 1.

Nowadays, diverse analytic methods were available for SHXXT quantitation, including micellar electrokinetic chromatography (MEKC) using silica capillary with UV detector for analysis of a mixture of coptis alkaloids, scute flavonoids, and rhubarb anthraquinones and bianthrones (Chang and Sun, 2006), HPLC-UV techniques combined with reverse-phase column eluted with organic solvent mixture with different buffer solutions (sodium 1-pentanesulphate and phosphorous acid) to detect several chemical markers in SHXXT (Huang et al., 2006; Li and Xu, 2006; Li Y. et al., 2010) as well as the UPLC-MS or LC-MS/MS instrument to separate and identify the bioactive ingredients utilizing C18 column and the mobile phase of acetonitrile mixed with ammonium acetate (Li S. L. et al., 2010; Zan et al., 2011). These methods suffer from several disadvantages, case by case, such as the limited sensitivity, longer analysis time, expensive instrument, lower resolution, or poor separation. To our knowledge, studies on either extensive chemical comparison between commercial SHXXT products or divergence quality analysis among *R. officinale* and *R. tanguticum* containing formulas have not been reported.

To establish a convenient HPLC-based chemical profiling method in order to obtain reliable and rapid separation of the major bioactive constituents of SHXXT was quite an important issue. After this work having been done, the connections among quantities of the nine internal bioactive components of commercial SHXXTs could directly be linked with the use of dosage, estimated pharmacodynamic effects to consumers, the predicted pharmacological mechanism and even based on the SAR (structure and activity relationship). The optimized HPLC analytical conditions were further successfully applied to the quantitative analysis of the bioactive constituents of 10 commercial SHXXT products, including traditional decoctions and dispensing granules.

## Materials and methods

### Chemical and materials

All chemicals were of analytical grade. Aloe-emodin (**Ale**), ammonium acetate, baicalin (**Ba**), berberine (**Be**), coptisine (**Co**), palmatine (**Pa**), resveratroloside (**Res**), rhein (**Rh**), sennoside A (**Se-A**), and wogonin (**Wo**) (≥98%) were purchased from Sigma-Aldrich (Sigma-Aldrich Corp., St. Louis, MO, USA). Formic acid and acetic acid (glacial, baker analyzed) was obtained from J. T. Baker (JT Baker Chemical Co., Phillipsburg, NJ, USA). Methanol and acetonitrile (HPLC grade) were purchased from Merck (Merck KGaA, Darmstadt, Germany). Deionized water (dd-H_2_O) was prepared using Milli-Q water purification system (Millipore, Bedford, MA, USA), which was used to prepare the buffers and related aqueous solutions. Nylon membrane filter (0.22 μm) was purchased from Millipore (Millipore, Bedford, MA, USA).

### HPLC-PDA conditions

A Shimadzu HPLC system with LC-20AD pump, SIL-20HT autosampler, SPD-M10A diode array detector was used. The signals of the UV detector (230, 254, 280, 340, 430 nm) were recorded and integrated by Shimadzu “Class VP” software. An Agilent Poroshell 120 HPLC column (Agilent Technologies, Inc., USA, 150 × 4.6 mm I.D., 2.7 μm) was utilized with the mobile phase of acetonitrile and aqueous solution with 4 mM ammonium acetate (pH 3.5, adjusted by formic acid) in a gradient system. The analysis started with 22% acetonitrile and increase to 24% acetonitrile in 3 min. Then acetonitrile was slowly increased to 25% at 9 min and increased to 28% acetonitrile at 10 min. This percentage of acetonitrile was increased quickly to 49% acetonitrile to 14 min and maintained for 7 min. At 21–25 min, the mobile phase was changed to 52.5% acetonitrile from 49% acetonitrile. Finally, acetonitrile was increased to 60% acetonitrile at 30 min. The flow rate used was 0.6 mL/min and the injection volume was 10 μL. The analysis was detected at 254 nm wavelength.

### LC-MS conditions

Qualitative LC-MS was performed using an Agilent 1100 HPLC system (Agilent Technologies) coupled to a Finnigan LCQ Deca XP Plus ion trap mass spectrometer (Thermo, Palo Alto, CA, USA). Besides replacing the flow rate of 0.6 by 0.5 mL/min, the instrumentation and chromatographic condition of HPLC for LC-MS were the same as the described in Section HPLC-PDA Conditions. The electrospray ionization (ESI) interface was used for analytical detection. ESI was applied in the positive and negative ionization modes and the capillary was held at a potential of 3.0 kV. The cone voltage was set at 40 V and the ionization source was set to a temperature of 350⋅C. Qualitative analysis was performed by monitoring [M + H]^+^ or [M − H]^−^ for analytes in selected ion recording mode and the mass range was set at m/z 100–1000, for identification purposes. All data acquisition and processing were performed using Xcalibur 2.0 software (Thermo, Palo Alto, CA, USA).

### HPLC method validation for standard analysis

To establish a standard quality control protocol, a specific calibration linear relationship was developed. The nine standards (1.00 mg) were prepared with 1 mL methanol, then filter by Nylon membrane filter (0.22 μm). The solution was further analysis by HPLC. For the preparation of the calibration curve, five standard concentrations of standard solutions (**Ale**: 0.075, 0.150, 0.300, 0.600, 1.200 μg/mL; **Ba**: 5.0, 10.0, 20.0, 40.0, 80.0 μg/mL; **Be**: 0.75, 1.50, 3.00, 6.00, 12.00 μg/mL; **Co**: 0.15, 0.30, 0.60, 1.20, 2.40 μg/mL; **Pa**: 0.25, 0.50, 1.00, 2.00, 4.00 μg/mL; **Res**: 0.75, 1.50, 3.00, 6.00, 12.00 μg/mL; **Rh**: 0.10, 0.20, 0.40, 0.80, 1.60 μg/mL; **Se-A**: 0.40, 0.80, 1.60, 3.20, 6.40 μg/mL; **Wo**: 0.0025, 0.0500, 0.1500, 0.3000, 0.6000 μg/mL) were prepared and quantitated by injecting each concentration three times on three consecutive days (*n* = 3). The limit of detection was determined by gradually decreasing concentration of the standard until signal to noise (S/N) ratio was 3. LOD and LOQ of nine standards are **Ale**: 0.01 μg/mL and 0.03 μg/mL, **Ba**: 0.18 μg/mL and 0.55 μg/mL, **Be**: 0.05 μg/mL and 0.16 μg/mL, **Co**: 0.02 μg/mL and 0.06 μg/mL, **Pa**: 0.01 μg/mL and 0.04 μg/mL, **Res**: 0.02 μg/mL and 0.06 μg/mL, **Rh**: 0.01 μg/mL and 0.02 μg/mL, **Se-A**: 0.03 μg/mL and 0.11 μg/mL, and **Wo**: 0.01 μg/mL and 0.02 μg/mL, respectively. The calibration curves were successfully constructed using the peak area of the standard (*y* axis) and the concentration of standard (μg/mL; *x* axis). Three concentrations (μg/mL) in three replicate injections were chosen to further investigate the intra- and inter-day precision and accuracy analysis over a sequence of 3 days (*n* = 3). Certain acceptance limits were developed for precision, linearity, and accuracy. For precision, it was established to be < 5% at RSD (%) of the peak area of three replicate injections. The linearity limits were set at correlation coefficient values exceeding 0.99. An accuracy value between 95 and 105% was considered acceptable for the intended purpose of this approach.

### Preparation of SHXXT samples

The prior-scale extraction product SHXXT-3 were chosen for the evaluation of the optimization of the extraction approaches of active components from commercial SHXXT products (SHXXT-1~10). Weighted 5.00 mg SHXXT-3 and dissolved in different methanol aqueous solutions (5, 50, and 100%) to obtain 0.50 mg/mL solutions. The test solutions were ultrasonicated for 30 min and the individual test solution was filtered with a 0.22-μm Millipore filter (Nylon membrane filter), three samples were prepared (10 μL aliquot) for further HPLC analysis (*n* = 3).

### Application of the developed HPLC protocol for commercial SHXXT products

The prior-scale extraction products SHXXT-1~3 (without additives, such as excipients, antiadherents, etc.) were purchased from one cGMP pharmaceutical company, and SHXXT-4~10 from seven different cGMP pharmaceutical companies in several different medicinal remedies (concentrated granules, powders, tablets etc. with ca. 50% additives, such as excipients, antiadherents, etc.). The herbal materials of those three Chinese medicines must be imported from the most typical local region of China, and each batch of them was selectively examined and validated for authorizing the national criterion of Ministry of Health and Welfare, Taiwan. The ratio of the active crude TCM (Dahuang:Huangqin:Huanglian = 2:1:1 or 1:1:1), and the additives were varied. It led to the quality examination of each product is difficult to control.

To prepare the samples (SHXXT-1~10) for analysis, weighted 5.00 mg samples and samples were dissolved in 50% methanol aqueous solution (10 mL). The sample solutions were ultrasonicated for 30 min, and centrifuged for 15 min (3000 rpm). The supernatant was filtered with a Nylon membrane filter (0.22 μm). One milliliter stock solution was diluted to 3 mL and a sample was taken from this diluted solution (10 μL aliquot) for the subsequent HPLC analysis. All the analyses were carried out and repeated for three times, and the data were recorded and expressed in concentration (μg/mL) of standard as mean ± *SD*.

## Results and discussion

HPLC equipped with UV-Visible detector and diode array detection (DAD) is the most common tool to qualitative and quantitative analysis of TCM because of its precision and accuracy. However, several parameters should be evaluated and optimized including the mobile phase, detection wavelength, and gradient elution method based on the peak area in the resulting HPLC chromatogram.

Previous report revealed that the best mobile phase solvents for HPLC analysis of *R. palmatum* (Dahuang), *S. Baicalensis* (Huangqin), *C. chinensis* (Huanglian), and SHXXT were methanol and acetonitrile (Li S. L. et al., 2010; Boyle et al., 2011; Wang G. Y. et al., 2011). For better stability, symmetric peaks and good resolution, combination with 0.1−0.5% organic acid, such as acetic acid, formic acid, trifluoroacetic acid, and phosphoric acid, in aqueous phase was used to balance the effect of the carboxylic group in **Ba**, **Rh**, and **Se-A** (Huang et al., 2006; Ren et al., 2007; Li et al., 2011). Dissociation constants between 3.4 and 3.6 were calculated using SPARC, an on-line pKa calculator to predict the dissociation constant of those acidic carboxylic group. Considering the broad application in industry and convenient preparations, reversed C-18 stationary phase was selected.

### Optimization of HPLC

**Figure 2:**
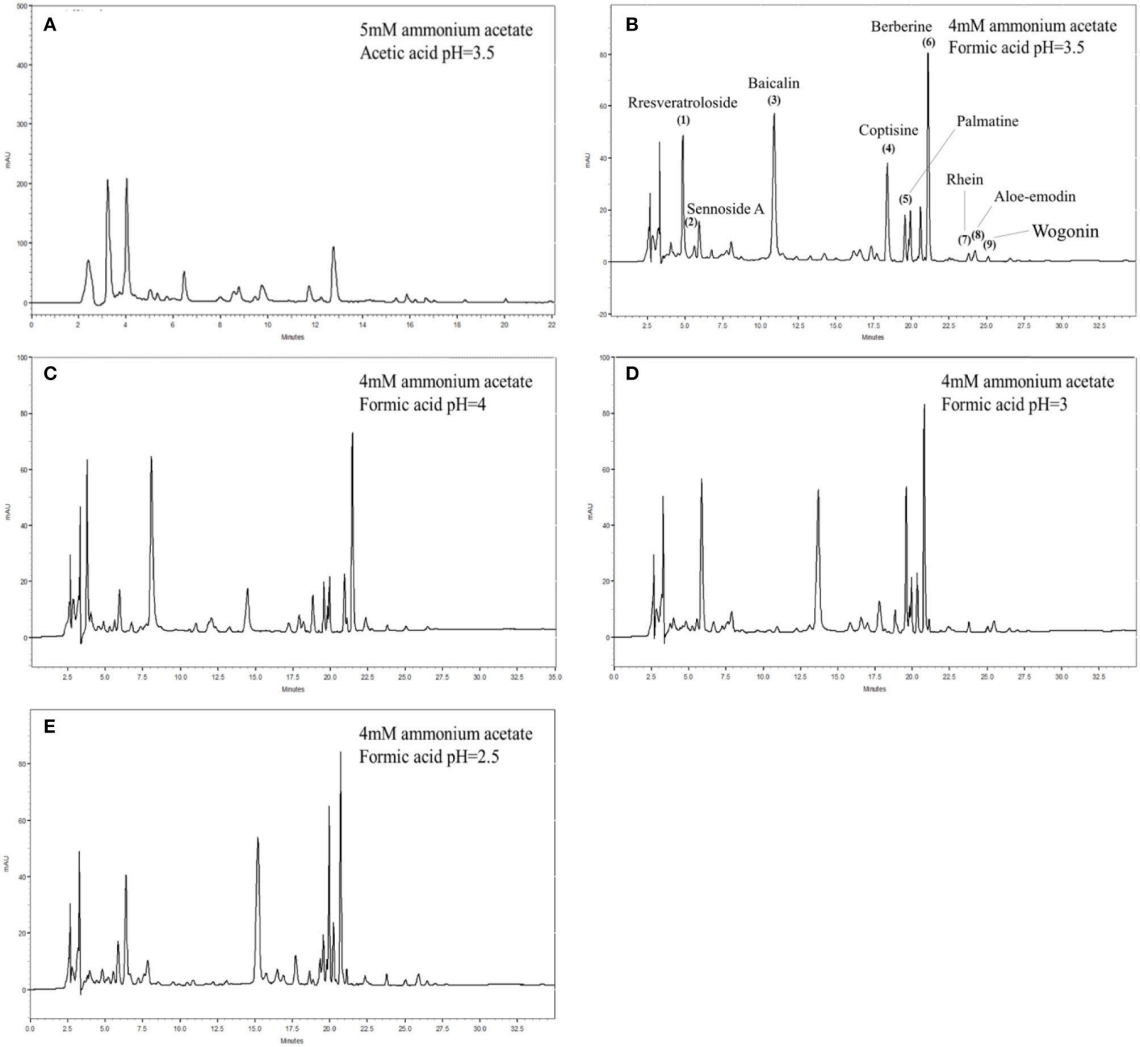
**Effects of ammonium acetate and buffer pH on the migration of analytes (0.50 μg/mL each)**. HPLC profiles of **(A)** 5 mM ammonium acetate and buffer pH 3.5, **(B)** 4 mM ammonium acetate and buffer pH 3.5, **(C)** 4 mM ammonium acetate and buffer pH 2.5, **(D)** 4 mM ammonium acetate and buffer pH 3.0, **(E)** 4 mM ammonium acetate and buffer pH 4.0. Peaks: 1, resveratroloside (**Res**); 2, sennoside A (**Se-A**); 3, baicalin (**Ba**); 4, coptisine (**Co**); 5, palmatine (**Pa**); 6, berberine (**Be**); 7, rhein (**Rh**); 8, aloe-emodin (**Ale**); 9, wogonin (**Wo**). HPLC condition in different ammonium acetate and buffer pH values: column, Agilent Poroshell 120 HPLC column (Agilent Technologies, Inc., USA, 150 × 4.6 mm I.D., 2.7 μm); mobile phase: an acetonitrile acetic-acetate buffer (4~5 mM; H_2_O, pH 2.5~4), 2:98 (v/v); flow rate, 0.6 mL/min; injection volume, 10 μL; UV detection: λ: 254 nm.

For optimization of the mobile phase, acetonitrile, and aqueous solution with 4 mM ammonium acetate, pH 3.5 (adjusted by acetic acid), and pH 2.5, 3.0, 3.5, 4.0 (adjusted by formic acid), as well as aqueous solution with 5 mM ammonium acetate, pH 3.5 (adjusted by acetic acid) were examined in this study. The results showed that the resolution of HPLC chromatogram while using 4 and 5 mM ammonium acetate in pH 3.5 (adjusted by acetic acid) were not optimal, because the peaks were broad and high salt concentration (5 mM) blocked the instrument, respectively. Thus, the formic acid was used instead of acetic acid to adjust the pH-value. In the Figure 2, different pH-values (2.5, 3.0, 3.5, and 4.0) adjusted by formic acid were tested, and the best resolution, and minimum asymmetry values were observed at pH 3.5.

**Figure 3:**
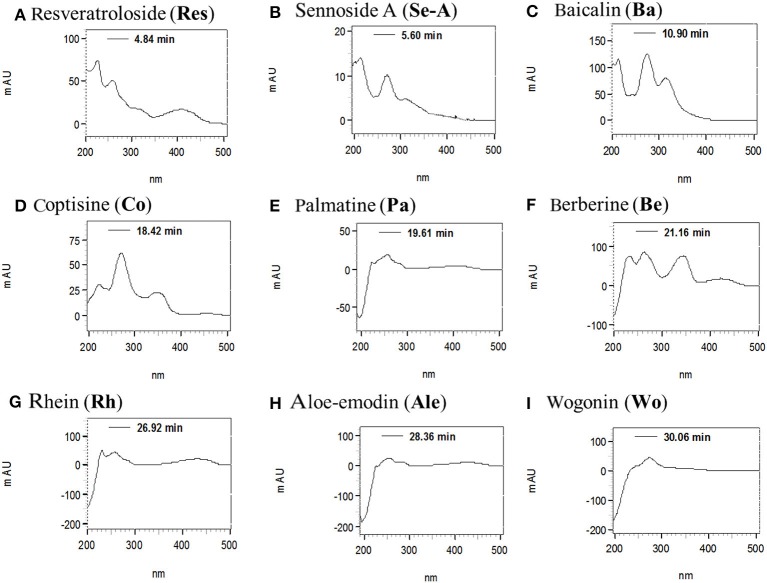
**UV spectra of nine chemical analytes in SHXXT. (A)** Resveratroloside (**Res**), **(B)** sennoside A (**Se-A**), **(C)** baicalin (**Ba**), **(D)** coptisine (**Co**), **(E)** palmatine (**Pa**), **(F)** berberine (**Be**), **(G)** rhein (**Rh**), **(H)** aloe-emodin (**Ale**), **(I)** wogonin (**Wo**).

**Figure 4:**
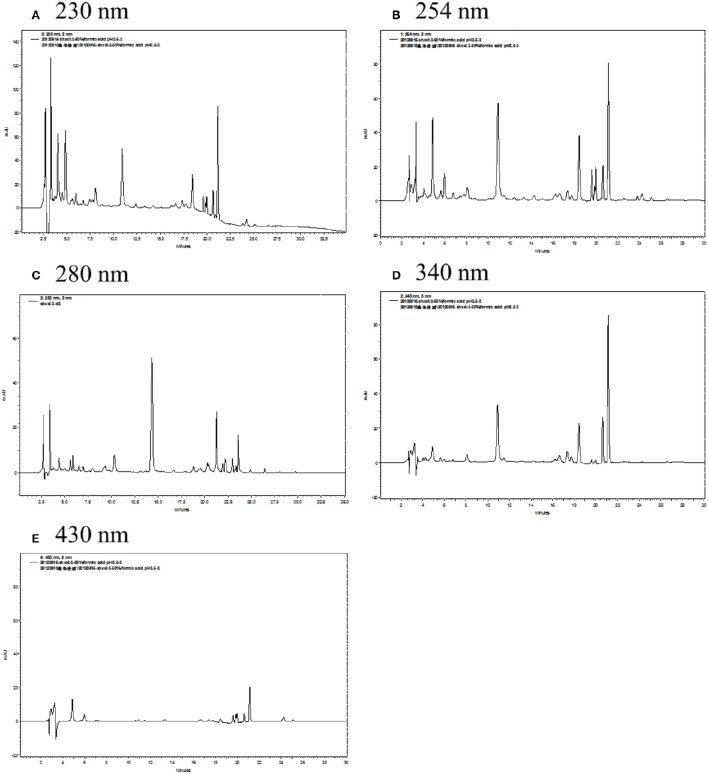
**Effects of five different UV detection wavelengths on the migration of analytes (0.50 μg/mL each)**. HPLC profiles of **(A)** 230 nm, **(B)** 254 nm, **(C)** 280 nm, **(D)** 340 nm, **(E)** 430 nm were arranged.

The structures of target anthraquinones, stilbenes, flavonoids, and alkaloids are characterized by conjugated double bonds and benzene rings, which implied the UV detectability. By comparison of UV spectra with those of reference compounds, the UV absorption of anthraquinones, flavonoids, stilbenes, and alkaloids were detected at 250–280 nm (Wu et al., 2004; Kong et al., 2009; Li et al., 2009; Li J. et al., 2010). The maxima of UV absorptions for nine target compounds were co-eluted and detected by photodiode array detector. Among them, the anthraquinones of **Se**-**A**, **Rh**, and **Ale** were recorded at 223, 273, 262, and 256 nm, respectively. The UV spectra of **Res** (stilbenes) had two absorbance maxima of 236 and 305 nm. The absorbance maxima of **Ba** and **Wo** (flavonoids) were found at 276, 317, and 275 nm. Otherwise, the maxima of UV absorptions of **Co**, **Pa**, and **Be** (alkaloids) were shown at 266, 273, and 262 nm, respectively (Figure 3). The UV spectra were compared at five different wavelengths (230, 254, 280, 340, and 430 nm) in this study (Figure 4). The UV results indicated that peak intensity of nine target compounds obtained under wavelengths of 254 nm. On the other hand, the nine target compounds can be detected in this particular wavelength by HPLC analysis in simultaneously. Therefore, 254 nm was selected as the suitable wavelengths.

However, the complicate ingredients in SHXXT led to the difficulties in the determination of the qualitative and quantitative analysis. The gradient elution of the mobile phase method was previously reported and referenced (Huang et al., 2006; Li S. L. et al., 2010; Li Y. et al., 2010). Acetonitrile and 4 mM ammonium acetate in pH 3.5 (adjusted by formic acid) were proposed and utilized in five different gradient elution programs as follows:

Program I: Time (solution A %): *t*_*R*_ 0.01−5 min (5−10%), 5−6 min (10−15%), 6−25 min (15%), 25−35 min (15−20%), 35−45 min (20%), 45−55 min (20−25%), 55−60 min (25−90%), 60−80 min (90%).
Program II: Time (solution A %): *t*_*R*_ 0.01−7 min (20−22%), 7−10 min (22−85%), 10−15 min (85−95%), 15−23 min (95%), 23−25 min (95−35%).
Program III: Time (solution A %): *t*_*R*_ 0.01−5 min (22−35%), 5−10 min (35−65%), 10−16 min (65−25%), 16−25 min (62−70%), 25−30 min (70−35%).
Program IV: Time (solution A %): *t*_*R*_ 0.01−3 min (22−24%), 3−10 min (24−27%), 10−14 min (27−50%), 14−17 min (50−52%), 17−30 min (52−60%).
Program V: Time (solution A %): *t*_*R*_ 0.01−3 min (22−24%), 3−9 min (24−25%), 9−10 min (25−58%), 10−13 min (28−49), 14−21 min (49%), 21−25 min (49−52.5%), 25−30 min (52.5−60%).

**Figure 5:**
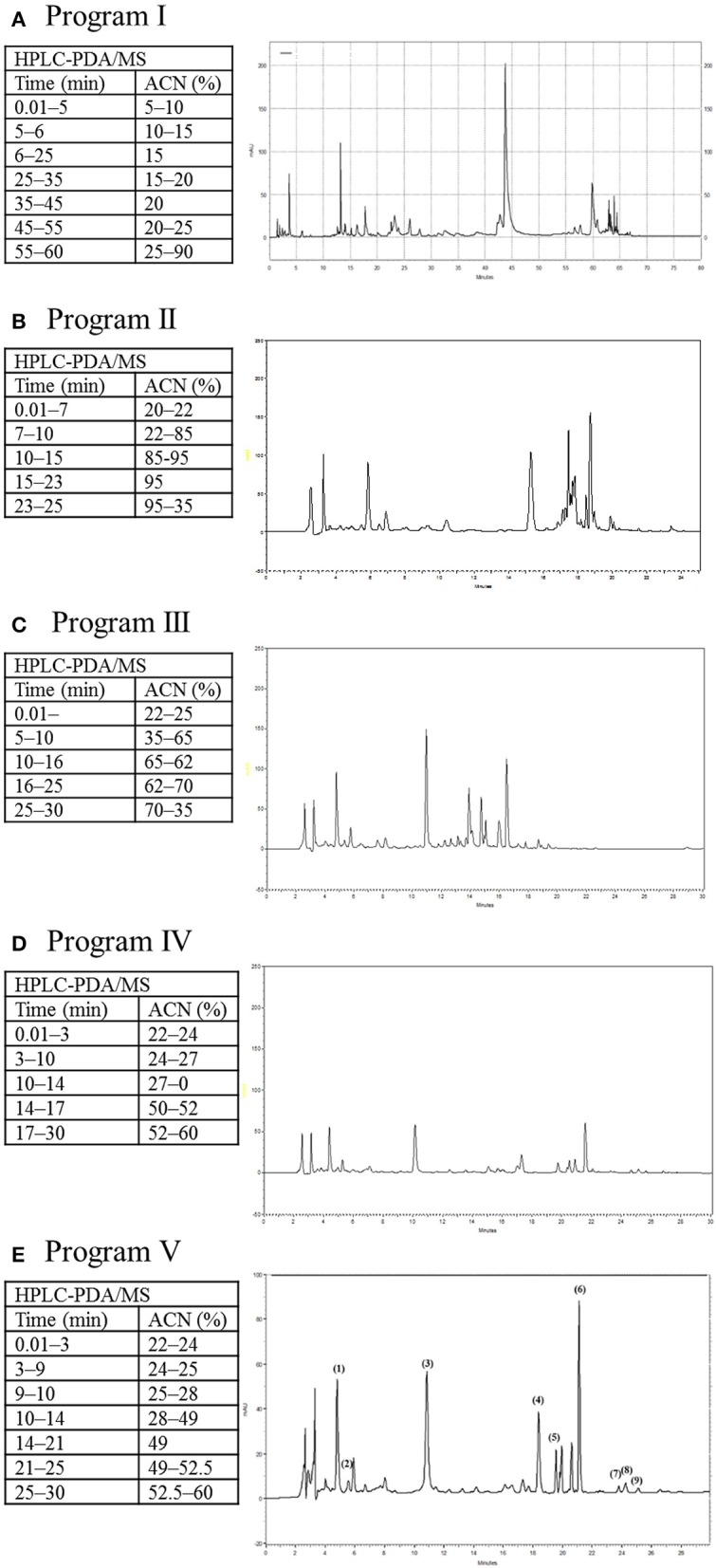
**Effects of five different gradient elution programs on the migration of analytes (0.50 μg/mL each)**. HPLC profiles of **(A)** Program I, **(B)** Program II, **(C)** Program III, **(D)** Program IV, **(E)** Program V were demonstrated.

As a result, Program V demonstrated the most well-defined peaks of the target compounds (Figure 5). Thus, the optimum HPLC condition is using the reversed phase HPLC, and a gradient elution with 4 mM ammonium acetate in pH 3.5 (adjusted by formic acid) at 0.6 mL/min which resulted in a good resolution and short analysis time (30 mins). The typical chromatogram (Figure 5E) indicated nine target compounds in a time-flow order: **Res** (*t*_*R*_ 4.83 min), **Se-A** (*t*_*R*_ 5.58 min), **Ba** (*t*_*R*_ 10.91 min), **Co** (*t*_*R*_ 18.42 min), **Pa** (*t*_*R*_ 19.58 min), **Be** (*t*_*R*_ 21.13 min), **Rh** (*t*_*R*_ 23.81 min), **Ale** (*t*_*R*_ 24.37 min), and **Wo** (*t*_*R*_ 25.12 min).

### Mass spectral analysis of SHXXT

**Figure 6:**
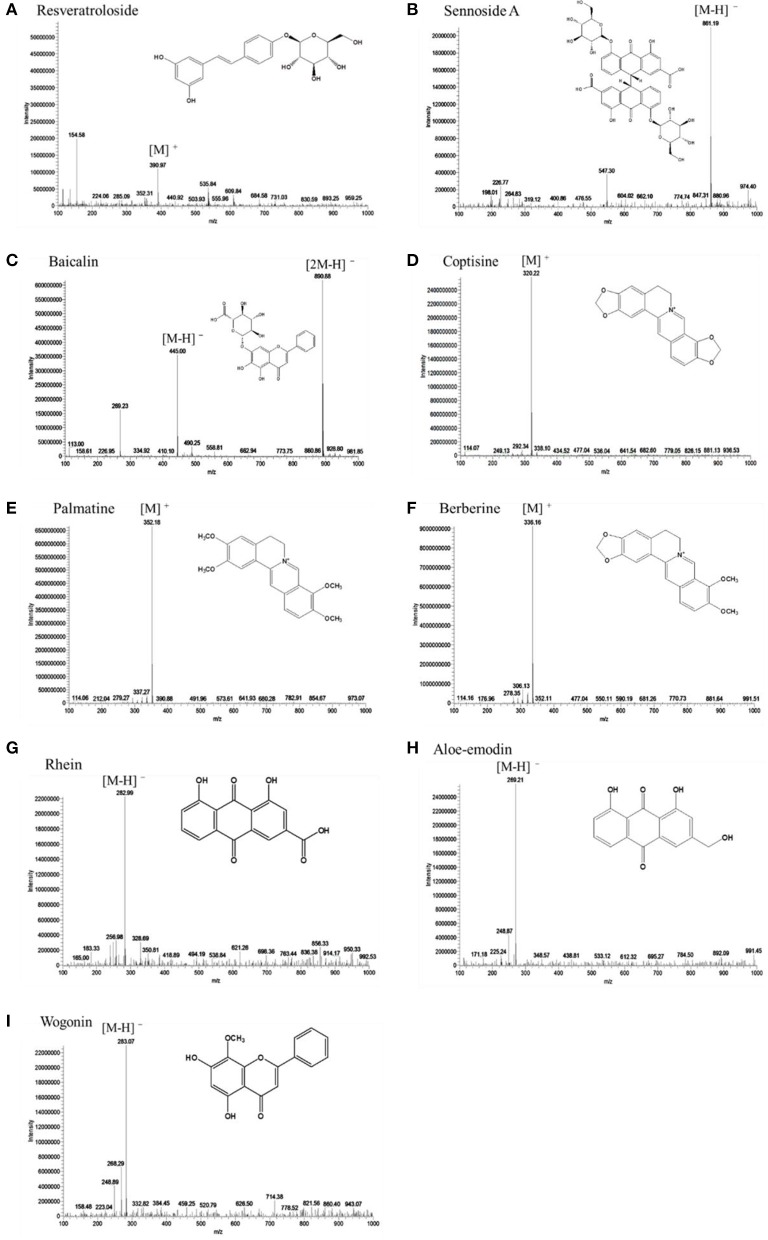
**LC-MS spectra of nine chemical analytes in SHXXT**. **(A)** resveratroloside—ESI(+), **(B)** sennoside A—ESI(–), **(C)** baicalin—ESI(–), **(D)** coptisine—ESI(+), **(E)** palmatine—ESI(+), **(F)** berberine—ESI(+), **(G)** rhein—ESI(–), **(H)** aloe-emodin—ESI(–), and **(I)** wogonin—ESI(–) were shown.

In order to confirm the purity of nine target compounds, the qualitative protocol was performed using HPLC coupled to an ion trap mass spectrometer and monitoring [M + H]^+^ or [M − H]^−^ for analytes in selected ion recording mode. The same developed HPLC method for analysis of SHXXT was also applied in LC-MS technique. Target compounds were prepared at 0.50 mg/mL and injected to LC-MS for further analysis. The LC-MS spectral was showed that four major types of compounds, including anthraquinones, flavonoids, stilbenes, and alkaloids were all detected and identified. Anthraquinones and flavonoids were detected in negative mode, while stilbenes and alkaloids were detected in positive mode (Figure 6). The target compounds of **Ale** (m/z 269.21 [M − H]^−^), **Ba** (m/z 890.88 [2M − H]^+^), **Rh** (m/z 862.99 [M − H]^−^), **Se-A** (m/z 861.19 [M − H]^−^), and **Wo** (m/z 283.07 [M − H]^−^) detected in negative mode and confirmed by comparison of UV/mass spectra with those of reference compounds. Identically, **Be** (m/z 336.16 [M]^+^), **Co** (m/z 320.22 [M]^+^), **Pa** (m/z 352.18 [M]^+^), and **Res** (m/z 390.97 [M]^+^) were observed in positive mode and confirmed by comparison of UV/mass spectra with those of reference compounds (Li S. L. et al., 2010). These results suggested that the developed HPLC method is able to analyze the bioactive compounds of SHXXT in a short time. Also, it revealed the good resolution and reproducibility of this developed method.

### Optimization of the extraction of SHXXT

**Figure 7:**
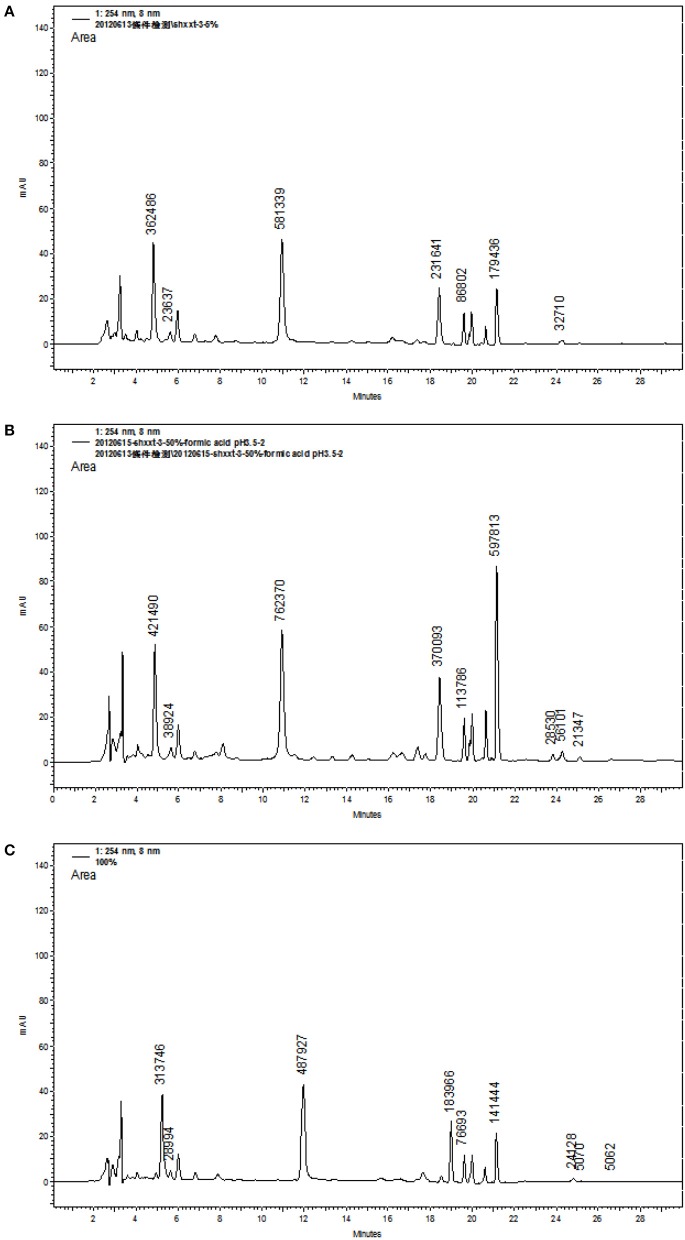
**HPLC profiles of SHXXT-3 in three different extraction methods, (A) 5% MeOH aqueous solution, (B) 50% MeOH aqueous solution, and (C) 100% MeOH aqueous solution**.

**Figure 8:**
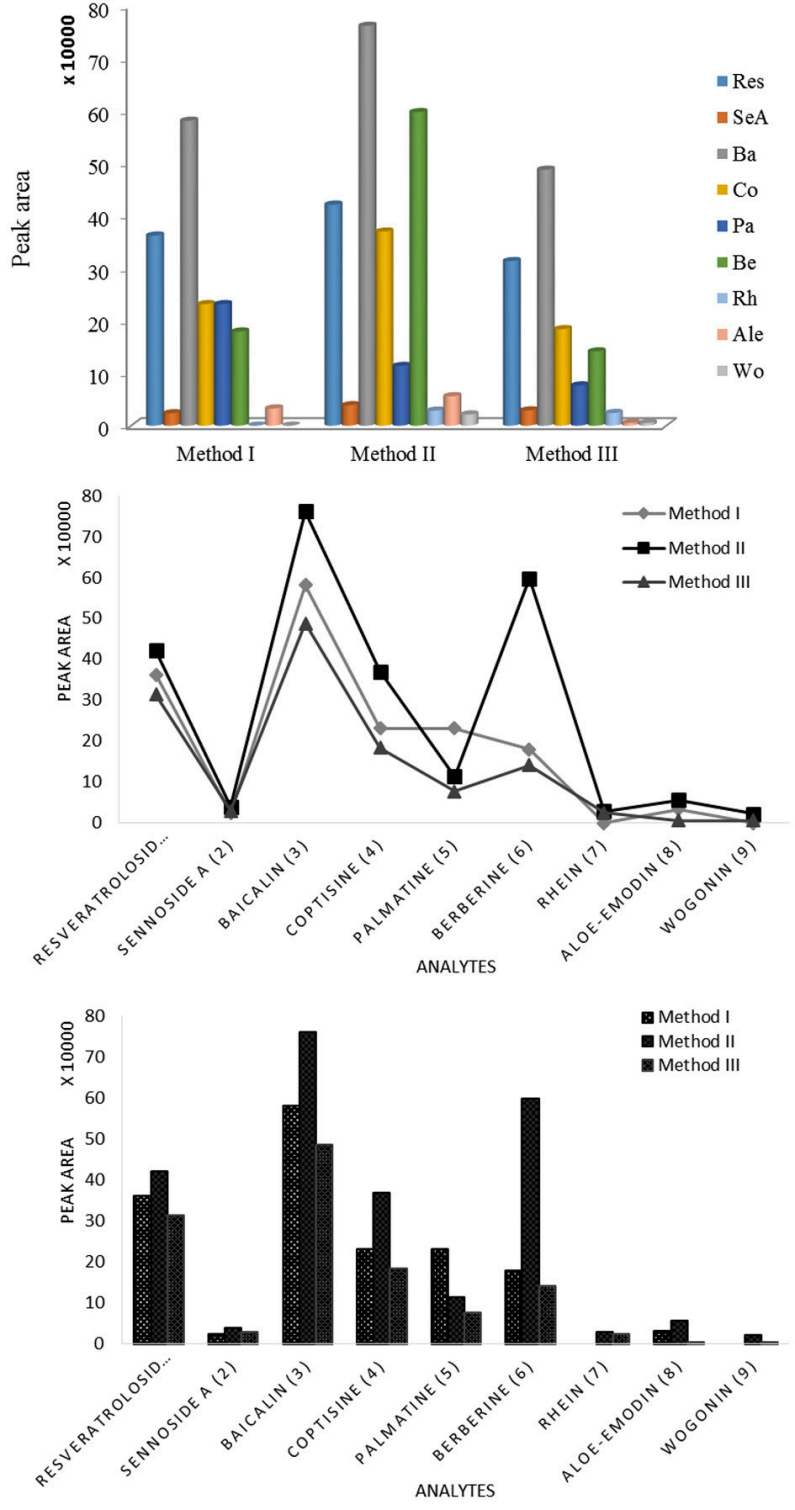
**Yields (%) of nine chemical markers isolated from SHXXT-3 by three different extraction methods**. Method I: 5% MeOH aqueous solution, Method II: 50% MeOH aqueous solution, Method III: 100% MeOH aqueous solution.

In order to concentrate the nine bioactive compounds in one fraction and make sure each fraction must contain the nine compounds, the samples of SHXXT-3 were extracted by 5, 50, and 100% MeOH_(*aq*)_ (Figure 7). In Figure 7, it indicated that the 50% MeOH_(*aq*)_ extract of SHXXT processing higher amount of nine bioactive compounds, such as **Ba** and **Be** (Figure 8). Baicalin (**Ba**) is the most abundant flavonoid in SHXXT, which result is similar to the previous report (Shih et al., 2007).

### Calibration, precision, and accuracy

The quantitative analysis of target compounds was evaluated and optimized. The quantitation range of each compound was: **Ale** (0.075−1.200 μg/mL), **Ba** (5.0−80.0 μg/mL), **Be** (0.75−12.00 μg/mL), **Co** (0.15−2.40 μg/mL), **Pa** (0.25−4.00 μg/mL), **Res** (0.75−12.00 μg/mL), **Rh** (0.10−1.60 μg/mL), **Se-A** (0.40−6.40 μg/mL), and **Wo** (0.025−0.600 μg/mL; Table 1). Five different concentrations of each compound were analyzed and the peak area was calculated. The calibration graph was established based on the peak area as the ordinate (Y) and the concentration (μg/mL) of target compound as the abscissa (X). Calculated linear regression equation of nine bioactive compounds was as following: **Ale** (Y = 75638.0X + 1326.2), **Ba** (Y = 37233.0X + 52341), **Be** (Y = 73590.0X − 1532.2), **Co** (Y = 55585.0X + 5375.3), **Pa** (Y = 13039.0X + 2777.9), **Res** (Y = 13280.0X + 3543.1), **Rh** (Y = 99814.0X + 8013.8), **Se-A** (Y = 42404.0X + 3029.2), and **Wo** (Y = 90308.0X + 2655.5) with the correlation coefficient (*R*^2^) reaching 0.99 (Table 1). The analysis method was a triplet repeat (*n* = 3). The limit of detection (LOD) of nine bioactive compounds was between 0.01 and 0.18 μg/mL and the limit of qualified (LOQ) of nine bioactive compounds was between 0.02 and 0.55 μg/mL (Table 2). This developed protocol has enhanced the sensitivity of the previous research (LOD: 0.17−2.06 μg/mL; Chang and Sun, 2006).

**Table 1:** **Regression analysis for the determination of nine analytes in intra-day and inter-day analysis^#^**.

**Compound (Peak No.)**	**Retention time (min)**	**Calibration range (μg/mL)**	**Regression equation**	**Coefficient of correlation (*R*^2^)**
Resveratroloside (**Res**, 1)	4.83	0.75−12.00	Y = 13280.0X + 3543.1	0.9931
Sennoside A (**Se-A**, 2)	5.58	0.40−6.40	Y = 42404.0X + 3029.2	0.9971
Baicalin (**Ba**, 3)	10.91	5.00−80.00	Y = 37233.0X + 52341	0.9983
Coptisine (**Co**, 4)	18.42	0.10−2.40	Y = 55585.0X + 5375.3	0.9903
Palmatine (**Pa**, 5)	19.58	0.25−4.00	Y = 13039.0X + 2777.9	0.9916
Berberine (**Be**, 6)	21.13	0.75−12.00	Y = 73590.0X − 1532.2	0.9986
Rhein (**Rh**, 7)	23.81	0.10−1.60	Y = 99814.0X + 8013.8	0.9939
Aloe-emodin (**Ale**, 8)	24.37	0.075−1.200	Y = 75638.0X + 1326.2	0.9975
Wogonin (**Wo**, 9)	25.12	0.025−0.600	Y = 90308.0X + 2655.5	0.9954

# *The regression equations for inter-day analysis were calculated from the assay values of the prepared standards on three different days (n = 3)*.

**Table 2:** **LOD and LOQ of nine analytes^#^**.

**Compound (Peak No.)**	**LOD (μg/mL)**	**LOQ (μg/mL)**
Resveratroloside (**Res**, 1)	0.02	0.06
Sennoside A (**Se-A**, 2)	0.03	0.11
Baicalin (**Ba**, 3)	0.18	0.55
Coptisine (**Co**, 4)	0.02	0.07
Palmatine (**Pa**, 5)	0.01	0.04
Berberine (**Be**, 6)	0.05	0.16
Rhein (**Rh**, 7)	0.01	0.02
Aloe-emodin (**Ale**, 8)	0.01	0.03
Wogonin (**Wo**, 9)	0.01	0.02

# *LOD, limit of detection; LOQ, limit of quantification*.

In inter- and intra-day analysis of this method, the peak area at three different concentrations of each compound was investigated. The results exhibited that the precision (relative standard deviations, RSD) and accuracy (relative errors, RE) of intra-day analysis were 0.16−7.78 and −0.31−14.78%, respectively; However, in inter-day analysis were 0.03−9.69 and 0.21−14.44%, respectively (Table 3). The relative recoveries of each compound was in the range of 94.6–106.8% (Table 4).

**Table 3:** **Precision and accuracy for the determination of nine analytes in intra-day and inter-day^#^**.

**Compound (Peak No.)**	**Intra-day analysis (*n* = 3)**			**Inter-day analysis (*n* = 3)**			**Accuracy (%)**
	**Concentration known^a^ (μg/mL)**	**Concentration found (μg/mL)**	**R.S.D (%)**	**Concentration known^a^ (μg/mL)**	**Concentration found (μg/mL)**	**R.S.D (%)**	
**RESVERATROLOSIDE (RES, 1)**							
	3	3.11 ± 0.14	4.37	3.56	2.98 ± 0.01	0.34	−0.67
	6	6.89 ± 0.62	9.01	14.78	6.53 ± 0.04	0.62	8.78
	12	12.09 ± 0.25	2.10	0.72	12.17 ± 0.18	1.50	1.44
**SENNOSIDE A (SE-A, 2)**							
	1.6	1.49 ± 0.09	6.08	−6.67	1.70 ± 0.03	1.48	6.46
	3.2	3.23 ± 0.02	0.47	0.83	3.15 ± 0.01	0.32	−1.56
	6.4	6.38 ± 0.01	0.16	−0.31	6.46 ± 0.14	2.10	0.89
**BAICALIN (BA, 3)**							
	20	19.87 ± 0.12	0.58	−0.63	21.58 ± 0.14	0.66	7.29
	40	41.17 ± 0.31	0.76	2.92	40.30 ± 0.67	1.66	0.76
	80	80.71 ± 0.75	0.93	0.89	82.50 ± 0.02	0.03	2.57
**COPTISINE (CO, 4)**							
	0.6	0.52 ± 0.02	2.96	3.56	0.57 ± 0.01	1.75	5.00
	1.2	1.31 ± 0.04	3.19	14.78	1.25 ± 0.03	2.44	4.44
	2.4	2.36 ± 0.01	0.42	0.72	2.51 ± 0.12	4.71	4.72
**PALMATINE (PA, 5)**							
	1	0.90 ± 0.07	7.78	−6.67	1.07 ± 0.02	1.42	7.33
	2	2.09 ± 0.02	0.73	0.83	2.09 ± 0.14	6.78	4.33
	4	4.39 ± 0.30	6.83	−0.31	4.04 ± 0.06	1.51	1.00
**BERBERINE (BE, 6)**							
	3	3.25 ± 0.16	4.77	−0.63	3.06 ± 0.08	2.47	2.11
	6	6.09 ± 0.16	2.58	2.92	6.20 ± 0.07	3.28	3.28
	12	12.24 ± 0.02	0.17	0.89	12.08 ± 0.12	0.69	0.69
**RHEIN (RH, 7)**							
	0.4	0.41 ± 0.02	1.67	3.56	0.37 ± 0.01	1.55	−6.67
	0.8	0.81 ± 0.02	0.83	14.78	0.81 ± 0.01	1.23	1.25
	1.6	1.63 ± 0.01	1.87	0.72	1.60 ± 0.02	0.95	0.21
**ALOE-EMODIN (ALE, 8)**							
	0.3	0.29 ± 0.02	−4.44	−6.67	0.30 ± 0.01	1.95	−1.11
	0.6	0.64 ± 0.02	7.22	0.83	0.62 ± 0.01	1.61	3.33
	1.2	1.22 ± 0.01	1.67	−0.31	1.22 ± 0.02	1.64	1.67
**WOGONIN (WO, 9)**							
	0.15	0.17 ± 0.01	11.11	−0.63	0.13 ± 0.01	7.69	−13.33
	0.3	0.32 ± 0.01	7.78	2.92	0.34 ± 0.02	4.45	14.44
	0.6	0.58 ± 0.01	−2.78	0.89	0.61 ± 0.01	1.64	1.67

# *Intra-day assay variance was validated with three known different concentrations of analytes at five intervals at a single day and inter-day assay variance was validated with three known different concentrations of analytes during three successive days*.

**Table 4:** **Recovery test of nine analytes**.

**Compound (Peak No.) Concentration(μg/mL)**	**R.S.D (%)**	**Recovery (%) Mean ± *S.D***
**RESVERATROLOSIDE (RES, 1)**		
6.0	2.59	103.41 ± 2.68
12.0	0.12	99.91 ± 0.12
**SENNOSIDE A(SE-A, 2)**		
1.60	1.82	100.87 ± 1.84
3.20	3.77	100.59 ± 3.79
**BAICALIN (BA, 3)**		
20.0	2.14	106.87 ± 2.29
40.0	0.96	100.58 ± 0.97
**COPTISINE (CO, 4)**		
1.20	1.19	102.41 ± 1.22
2.40	5.52	101.33 ± 5.59
**PALMATINE (PA, 5)**		
2.0	2.10	102.95 ± 2.16
4.0	3.01	102.58 ± 3.09
**BERBERINE (BE, 6)**		
6.0	0.15	100.17 ± 0.15
12.0	0.59	100.86 ± 0.60
**RHEIN (RH, 7)**		
0.80	5.46	94.66 ± 5.17
1.60	0.28	100.40 ± 0.28
**ALOE-EMODIN (ALE, 8)**		
0.30	4.85	97.65 ± 4.73
1.20	4.10	97.58 ± 4.00
**WOGONIN (WO, 9)**		
0.30	1.91	97.56 ± 1.86
0.60	1.85	98.82 ± 1.82

### Application

Ten SHXXT products (SHXXT-1~10) were subjected to HPLC analysis to determine the bioactive composition by the developed method. The HPLC profiles for the sample SHXXT-3 can be applied to all test products. Table 5 listed the results of tested SHXXT products. The prior-scale extraction products SHXXT-1~3 showed higher chemical contents owing to without additives. In SHXXT-1~3, **Res** (>56.59%) and **Ba** (>27.91%) were found as the major compounds with significantly higher amounts twice than those in other products from other pharmaceutical companies (Table 5).

**Table 5:** **Analytical results of nine analytes in commercial SHXXT products^#^**.

**Compound (Peak No.)**	**SHXXT-1**		**SHXXT-2**		**SHXXT-3**		**SHXXT-4**		**SHXXT-5**		**SHXXT-6**		**SHXXT-7**		**SHXXT-8**		**SHXXT-9**		**SHXXT-10**	
	**Concentration found (μg/mL)**	**RSD (%)**	**Concentration found (μg/mL)**	**RSD (%)**	**Concentration found (μg/mL)**	**RSD (%)**	**Concentration found (μg/mL)**	**RSD (%)**	**Concentration found (μg/mL)**	**RSD (%)**	**Concentration found (μg/mL)**	**RSD (%)**	**Concentration found (μg/mL)**	**RSD (%)**	**Concentration found (μg/mL)**	**RSD (%)**	**Concentration found (μg/mL)**	**RSD (%)**	**Concentration found (μg/mL)**	**RSD (%)**
Resveratroloside (**Res**, 1)	56.59 ± 0.1	0.2	58.60 ± 0.4	0.6	61.69 ± 0.1	0.2	16.18 ± 0.1	0.5	26.92 ± 0.6	2.1	−	−	−	−	17.11 ± 0.2	1.1	−	−	−	−
Sennoside A (**Se-A**, 2)	5.78 ± 0.0	0.2	5.50 ± 0.0	0.3	5.64 ± 0.0	0.3	0.78 ± 0.0	1.0	0.95 ± 0.0	0.5	16.60 ± 0.1	0.5	8.28 ± 0.2	2.6	4.37 ± 0.1	0.3	2.41 ± 0.2	6.4	12.18 ± 0.1	0.5
Baicalin (**Ba**, 3)	27.91 ± 0.3	0.9	29.79 ± 0.2	0.7	34.71 ± 0.7	1.9	9.90 ± 0.1	0.9	10.28 ± 0.1	0.9	11.17 ± 0.1	0.8	4.15 ± 0.1	2.1	25.32 ± 0.1	0.4	11.11 ± 0.2	1.7	39.97 ± 0.1	0.1
Coptisine (**Co**, 4)	11.70 ± 0.1	0.8	11.64 ± 0.1	0.9	12.64 ± 0.1	1.1	6.89 ± 0.2	3.4	7.58 ± 0.0	0.6	8.02 ± 0.1	1.0	−	−	6.85 ± 0.1	0.8	0.88 ± 0.0	1.3	4.76 ± 0.1	1.4
Palmatine (**Pa**, 5)	18.22 ± 0.1	0.4	18.73 ± 0.1	0.6	21.65 ± 0.3	1.3	11.57 ± 0.0	0.3	9.78 ± 0.0	0.2	14.12 ± 0.2	1.6	18.73 ± 0.1	0.6	13.77 ± 0.2	1.3	0.73 ± 0.0	4.2	6.43 ± 0.1	1.6
Berberine (**Be**, 6)	8.64 ± 0.1	0.6	8.99 ± 0.1	1.1	12.85 ± 0.2	1.3	10.78 ± 0.0	0.7	9.60 ± 0.1	0.5	26.38 ± 0.2	0.6	5.68 ± 0.1	1.1	10.09 ± 0.2	1.7	4.33 ± 0.1	1.2	8.68 ± 0.2	1.8
Rhein (**Rh**, 7)	0.23 ± 0.0	4.4	0.57 ± 0.0	2.6	0.88 ± 0.0	0.4	0.04 ± 0.0	8.8	0.1 ± 0.0	3.3	−	−	0.57 ± 0.0	2.6	1.47 ± 0.0	1.6	0.04 ± 0.0	8.8	0.01 ± 0.0	5.7
Aloe-emodin (**Ale**, 8)	0.34 ± 0.0	11.0	0.16 ± 0.0	2.5	0.40 ± 0.0	1.0	0.17 ± 0.0	2.7	0.24 ± 0.0	2.6	1.2 ± 0.0	0.8	0.8 ± 0.0	0.7	0.9 ± 0.0	1.1	0.17 ± 0.0	2.7	0.23 ± 0.0	2.7
Wogonin (**Wo**, 9)	0.15 ± 0.0	3.0	0.27 ± 0.0	1.2	0.16 ± 0.0	2.3	0.16 ± 0.0	2.9	0.17 ± 0.0	0.3	0.7 ± 0.0	0.8	0.8 ± 0.0	3.0	0.8 ± 0.0	0.7	0.16 ± 0.0	2.9	0.17 ± 0.0	0.3

# *not detected*.

Moreover, the results showed that the contents of the chemical components in different commercial SHXXT product extracts were quite different. Interestingly, some products of companies did not have **Res**. We found that the phenomenon was due to the uses of different origins of Rheum, *R. tanguticum*, and *R. officinale*. While the companies used the raw material *R. tanguticum*, the formula will have **Res**. **Res** in Dahuang was rich in SHXXT-1~5 and 8 with 16.18−61.69% while **Ba** in Huangqin was abundant in SHXXT-1~3, 5, 9, and 10 with 10.28−39.97%. For the compositions of Huanglian, **Be** was abundant in SHXXT-6 (26.38%), and **Pa** was enriched in SHXXT-4, 7, and 8 with 11.57−18.73%. Simultaneously, **Rh**, **Ale**, and **Wo** were < 2% in these 10 products.

Ten commercial products showed different chemical profiles that indicated the importance to establish an easily and efficacy of the quality control analysis method for SHXXT.

Moreover, to analyze the raw materials of SHXXT from different regions to clarify the divergences for deciphering the parameters on CMC (chemical manufacturing control) and GAP (good agricultural practices) was also an indispensable job. However, the samples of SHXXT in this study were directly obtained from several CGMP-TCM biotechnical companies. The exact comprising ratio and sources of the three materials, rhizome of *R. officinale*, rhizome of *R. tanguticum*, root of *S. baicalensis*, and rhizome of *C. chinensis*, were even some kinds of know-how for them. The only information we could know was that the Chinese medicinal materials must had been imported from the most typical local region of China.

Attributed to the aforementioned, the established QC method in this work was created through the most representative SHXXT products in the Chinese-speaking world. Even this study was the first attempt for establishing a discriminative and accurate QC method for SHXXT, the comprehensive comparisons among those materials from different regions were an imperative subject to be exhaustively researched in the near future.

## Conclusion

A simple, efficient and reliable HPLC-PDA method was developed for the analysis of nine bioactive compounds in SHXXT. The method was associated with LC-MS technique for qualitative identification of active compounds in the tested samples. The fine extraction solution was identified and the optimized HPLC condition was validated in a good precision and accuracy. Furthermore, this protocol was successfully applied for the detection and quantification of 10 different SHXXT products. Thus, the development of this method can be applied to routine quality control analysis of SHXXT or Dahuang/Huangqin/Huanglian containing TCM products.

What are the reasons for different chemical compositions in producing SHXXT? We concluded the results owing to the follows:

1. The different qualities and origins of materials, such as *R. tanguticum*, and *R. officinale*, are applied to the manufacture.
2. Practically, in Taiwan, the government allows TCM pharmaceutical companies to apply a TCM license to manufacture according to the TCM literature. In the literature, they may use the ratios of 2:1:1 or 1:1:1 for the materials Dahuang:Huangqin:Huanglian to carry out the products.
3. The variety of SHXXT products may be caused by adding additives in different formulations. To overcome this problem, we suggest that the manufacturers shall use the additives NOT influence the composition of a formulated product.
4. Different factories may have at odds equipment and processes in manufacturing. For examples, different heating or evaporating equipment leads to inconsistent time consumption in the concentration of extracts. Standard chemical profiles lead to standard bioresponse. As results, since different chemical profiles of several commercial products were observed; therefore, probably, each same-named formula product might be regarded as a sole medicine and need to be investigated individually. We would like to recall the importance of quality control in TCM development.

## Author contributions

Substantial contributions to the conception or design of the work: TW, FC, YW, and CL. The acquisition, analysis: TW and LL. Interpretation of data for the work: TW, JL, IL, TC, LL. Drafting the work: JL, CC, YD, MW, and SJ. Revising it critically for important intellectual content: FC, JL, IL, YW, and CL. Final approval of the version to be published: FC, YW, and CL. Agreement to be accountable for all aspects of the work in ensuring that questions related to the accuracy: TW, FC, JL, IL, TC, LL, CC, YD, MW, SJ, YW, and CL. Integrity of any part of the work are appropriately investigated and resolved: TW, FC, JL, IL, TC, LL, CC, YD, MW, SJ, YW, and CL.

### Conflict of interest statement

The authors declare that the research was conducted in the absence of any commercial or financial relationships that could be construed as a potential conflict of interest.

## Abbreviations

SHXXT: San-Huang-Xie-Xin-Tang
TCM: traditional Chinese medicine
**Ale**: aloe-emodin
**Ba**: baicalin
**Be**: berberine
**Co**: coptisine
**Pa**: palmatine
**Res**: resveratroloside
**Rh**: rhein
**Se-A**: sennoside A
**Wo**: wogonin
**CGMP**: current good manufacturing practice.
